# Measurement and Analysis of Thermal Conductivity of Ti_3_C_2_T_x_ MXene Films

**DOI:** 10.3390/ma11091701

**Published:** 2018-09-13

**Authors:** Lin Chen, Xuguo Shi, Nanjie Yu, Xing Zhang, Xiaoze Du, Jun Lin

**Affiliations:** 1 Key Laboratory of Condition Monitoring and Control for Power Plant Equipment of Ministry of Education, North China Electric Power University, Beijing 102206, China; yunanjie@icloud.com (N.Y.); duxz@ncepu.edu.cn (X.D.); 2 Department of Engineering Mechanics, Tsinghua University, Beijing 100084, China; shixuguo1001@outlook.com (X.S.); x-zhang@tsinghua.edu.cn (X.Z.); 3 School of Renewable Energy, North China Electric Power University, Beijing 102206, China; 4 China United Engineering Corporation Limited, Hangzhou 310052, China

**Keywords:** thermal properties, electrical properties, MXene, T-type method

## Abstract

A new class of 2D materials named “MXene” has recently received significant research interest as they have demonstrated great potential for the applications in batteries, supercapacitors, and electronic devices. However, the research on their thermal properties is still very limited. In this work, Ti_3_C_2_T_x_ films were prepared by the vacuum-assisted filtration of delaminated nano-flake Ti_3_C_2_T_x_ MXenes. The thermal and electrical conductivity of the Ti_3_C_2_T_x_ films were measured by the state-of-the-art T-type method. The results showed that the effective thermal conductivity of the films increased from 1.26 W·m^−1^·K^−1^ at 80 K to 2.84 W·m^−1^·K^−1^ at 290 K, while the electrical conductivity remained at 12,800 Ω^−1^·m^−1^ for the same temperature range. Thermal resistance model was applied to evaluate the inherent thermal conductivity of the Ti_3_C_2_T_x_ flakes, which was estimated to be in the range of tens to hundreds W·m^−1^·K^−1^.

## 1. Introduction

The past decade has witnessed tremendous research efforts devoted to two-dimensional (2D) materials because they exhibit novel thermal, electrical, and mechanical properties as compared to their bulk materials. One of the most recent developments of 2D materials concerns a new class of 2D transition metal carbide and/or nitride named “MXene”, which is usually fabricated by selectively etching “A” from M_n+1_AX_n_ phases (M is an early transition metal, A is an A-group element, X is carbon and/or nitrogen, and n = 1, 2 or 3) [1]. Since M_n+1_AX_n_ phases consist of more than 60 ternary carbides and nitrides, there are significant opportunities to tune the properties of MXenes by changing their elemental composition [1,2]. To date, Ti_3_C_2_T_x_ (T refers to the surface-terminating functional groups) is one of the most studied MXenes, which is generally produced by the room temperature Al etching of Ti_3_AlC_2_ by hydrofluoric acid (HF). Owning to its excellent physical and chemical properties such as large elastic moduli, tunable bandgap, hydrophilic surface, high electrical conductivity and ductility [1,3], Ti_3_C_2_T_x_ nanocrystals (especially in forms of delaminated nanosheets/flakes) are expected to have potential applications in a wide range of energy storage devices including lithium-ion batteries and super capacitors (pseudo-capacitors). 

Lukatskaya et al. [3] demonstrated that both multilayer exfoliated Ti_3_C_2_T_x_ and MXene paper made of few layers of Ti_3_C_2_T_x_ could be intercalated by a variety of aqueous salt solutions. The resulting materials offered capacitance in excess of 300 F/cm^3^, and the highest volumetric capacitance of 350 F/cm^3^ was observed in NaOH solution for Ti_3_C_2_T_x_ paper, which is mainly attributed to the higher specific surface area of the Ti_3_C_2_T_x_ paper resulted from delamination. Mashtalir et al. [4] studied the MXene intercalation by hydrazine, urea and dimethyl sulfoxide (DMSO), respectively. They found that Ti_3_C_2_ intercalated by DMSO and delaminated by sonication in water formed a stable colloidal solution, which was filtered to produce MXene paper showing excellent Li-ion capacity. Ghidiu et al. [5] found a faster and safer route to delaminate Ti_3_C_2_ flakes, which yields clay-like materials. Rolled film of such Ti_3_C_2_ clay achieved volumetric capacitances up to 900 F/cm^3^, showing potential in electrochemical energy storage applications. The delaminated Ti_3_C_2_T_x_ can also be used as polymer composite fillers. Ling et al. [6] produced atomically thin Ti_3_C_2_T_x_ MXene by delamination and they prepared both pure Ti_3_C_2_T_x_ film and Ti_3_C_2_T_x_/polymer (poly(diallyldimethylammonium chloride) (PDDA) and polyvinyl alcohol (PVA)) composite films. The resulting flexible films achieved quite high electrical conductivities: 2.2 × 10^4^ S/m for Ti_3_C_2_T_x_/PVA composite and 2.4 × 10^5^ S/m for pure Ti_3_C_2_T_x_ film.

However, compared to the relatively extensive research on the electrical properties of Ti_3_C_2_T_x_, there is very limited study on its thermal properties. In fact, thermal conductivity is also essential for energy related applications. It is well known that higher thermal conductivity helps to achieve better heat dissipation, which is necessary to ensure the safety and reliability of batteries [7] and capacitors [8]. Although theoretical thermal conductivity values of several MXene materials (e.g., Hf_2_CO_2_, Sc_2_CT_2_) have been predicted [9], experimental results for Ti_3_C_2_T_x_ MXene still lack. Recently, Liu et al. [10] measured the thermal conductivity of Ti_3_C_2_T_x_ film and Ti_3_C_2_T_x_/PVA film by the laser flash technique. However, these films were made from un-delaminated multilayered Ti_3_C_2_T_x_, which is different from the delaminated Ti_3_C_2_T_x_ nano-flakes as used in the abovementioned literature. Therefore, the experimental results on the thermal conductivity of Ti_3_C_2_T_x_ films made from stacked delaminated nano-sheet/flake would be informative for the energy related applications.

In this work, Ti_3_C_2_T_x_ films were prepared by vacuum-assisted filtration of delaminated nano-flake Ti_3_C_2_T_x_ MXenes, which were produced by selective Al etching of Ti_3_AlC_2_ followed by DMSO intercalation and sonication-assisted delamination. Due to the relatively low thickness of the prepared film (tens μm), which is beyond the thickness requirement of commercially available laser flash instruments (at least 100 μm), the thermal and electrical conductivities of the prepared films were measured by the state-of-the-art T-type method, which was developed by Zhang et al. [11] to measure the thermal and electrical conductivities of carbon fiber and later extended to measure a wide range of micro/nanoscale samples [12,13,14,15,16]. The experimental results were also analyzed by using a simplified thermal resistance model to further understand the thermal performance of Ti_3_C_2_T_x_ films.

## 2. Materials and Methods 

### 2.1. Materials

Ti_3_AlC_2_ (98% purity) powders were bought from Forsman Scientific, Beijing, China. Hydrofluoric acid (HF, 40 wt%) and absolute ethyl alcohol were supplied by Beihua Fine Chemicals, Beijing, China. Dimethyl sulfoxide (DMSO) was produced by Fucheng, Tianjin, China. Polytetrafluoroethylene (PTFE) membrane (pore size 100 nm) was manufactured by Laisheng, Haining, China. Polypropylene (PP) membrane (Celgrad-2400, pore size 43 nm) was supplied by Celgard, Charlotte, NC, USA. All materials were used as received.

### 2.2. Preparation of Ti_3_C_2_T_x_ Powders

HF was used to etch the Al layer from Ti_3_AlC_2_ to obtain Ti_3_C_2_T_x_ according to the procedures described in the literature [17]. Briefly, Ti_3_AlC_2_ powders (2.5 g) were placed in a 100 mL plastic beaker and 60 mL HF (40 wt%) was slowly added to the plastic beaker, which was kept in the fume hood. The mixture was magnetically stirred at room temperature for 24 h. During this period, the Al was selectively etched, turning the Ti_3_AlC_2_ into multilayered Ti_3_C_2_T_x_. The mixture was diluted by deionized (DI) water and centrifuged at 4000 rpm to obtain the precipitate, which was washed repeatedly by DI water until the pH value of supernatant was 6. The aqueous dispersion of multilayered Ti_3_C_2_T_x_ was vacuum filtered by the PTFE membrane to obtain the filter cake, which was washed by absolute ethyl alcohol and then dried in air for 12 h.

### 2.3. Preparation of Ti_3_C_2_T_x_ Film 

Ti_3_C_2_T_x_ powders (1 g) were added to 12 mL DMSO and magnetically stirred at room temperature for 24 h. Then 30 mL DI water was added and the mixture was centrifuged at 3500 rpm for 5 min to obtain the precipitate, which was dispersed in 300 mL deoxygenated DI water. The suspension was then ultrasonicated for 7 h under argon, followed by the centrifugation at 3500 rpm for 1 h. The supernatant was collected and vacuum filtered by the porous PP membrane. After 6 h of air drying, the Ti_3_C_2_T_x_ film with a thickness of 10~20 μm was detached from the PP membrane. Strips of ~0.8 mm × 10 mm (width × length) were cut from the Ti_3_C_2_T_x_ film for the thermal and electrical conductivity measurements.

### 2.4. Characterization

The morphologies of Ti_3_AlC_2_ and Ti_3_C_2_T_x_ powders and Ti_3_C_2_T_x_ film were observed by scanning electron microscope (SEM, SU8010, Hitachi, Tokyo, Japan) with integrated Energy-dispersive X-ray spectroscopy (EDS, EX-350, Horiba, Kyoto, Japan) for element analysis. Both Ti_3_AlC_2_ and Ti_3_C_2_T_x_ powders were characterized by X-ray diffractometer (XRD, Cu/K-α1 radiation, Bruker D8, Karlsruhe, Germany) under 40 kV and 40 mA with a scanning speed of 5 °/min from 5° to 65°.

### 2.5. Measurements

**Figure 1:**
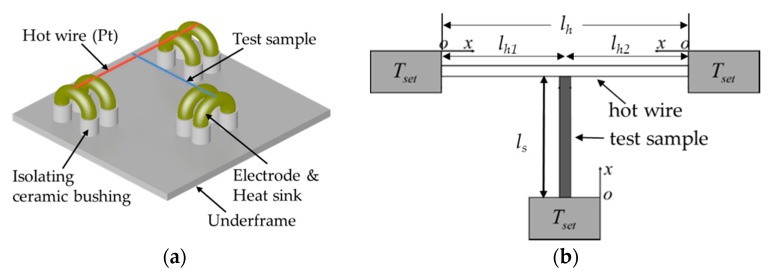
Schematic of the T-type method. (**a**) Connecting the test sample and the (**b**) physical model.

Thermal and electrical conductivities of Ti_3_C_2_T_x_ samples were measured by the steady-state T-type method as shown in Figure 1. Platinum (Pt) wire of 25 μm in diameter and ~32 mm in length, was welded at both ends to copper electrodes (heat sink) which were supported and isolated by ceramic bushings fixed on the metal underframe (Figure 1a). One end of the test sample was attached at the center of the Pt wire (i.e., hot wire) with silver gel and the other end was attached to the copper electrode. The underframe was placed in a vacuum chamber (Cryomech Incorporation, PT403, Syracuse, New York, NY, USA) to minimize the heat losses from convection and radiation. The pressure in the camber was kept lower than 10^−4^ Pa by using a mechanical pump (Leybold, R5614Y-Z, Cologne, Germany) and a molecular pump (Leybold, TW70H). The temperature of heat sink was controlled by a temperature controller (Oxford Instruments, ITC 503, Abingdon, England) and the temperature could be adjusted from 2.8 K to 300.0 K with an accuracy of 0.1 K using liquid helium evaporation and electric heater.

During measurements, a constant voltage source (Advantest, R6243, Sendai, Japan), a standard resistor (Yokogawa, 2792, Musashino, Japan), the hot wire, and the test sample composed the circuit. In the steady state T-type method for measuring thermal conductivity and the standard four-probe method for measuring electrical conductivity, the voltage drops were measured by two high-precision digital multimeters (Keithley, 2002, Beaverton, OR, USA). The detailed measurement principle and procedure are available in References [11,12,13,16], and the thermal conductivity of the sample *λ_s_* in this work is calculated by Reference [16].

(1) $$\lambda_{s}=\frac{L_{s}L_{h}\lambda_{h}A_{h}(L_{h}^{3}q_{V}-12L_{h}\lambda_{h}\Delta T_{V})}{L_{h1}L_{h2}A_{s}\left[12L_{h}\lambda_{h}\Delta T_{V}-q_{V}(L_{h1}^{3}+L_{h2}^{3})\right]}$$

where *L_s_* is length of test sample between two connecting ends, *L_h_* is the length of the hot wire, which equals to *L_h_*_1_ + *L_h_*_2_, as shown in Figure 1b. *A_h_* and *A_s_* are the cross-sectional areas of the hot wire and the test fiber, respectively. *λ_h_* is the thermal conductivity of platinum wire, *q_V_* = *UI*/*L_h_A_h_* is the volumetric heating power of the hot wire, and Δ*T_V_* is the volumetric average temperature rise of the hot wire.

## 3. Results and Discussion

### 3.1. Characterization of Ti_3_C_2_T_x_

#### 3.1.1. Ti_3_AlC_2_ and Ti_3_C_2_T_x_ Powders

**Figure 2:**
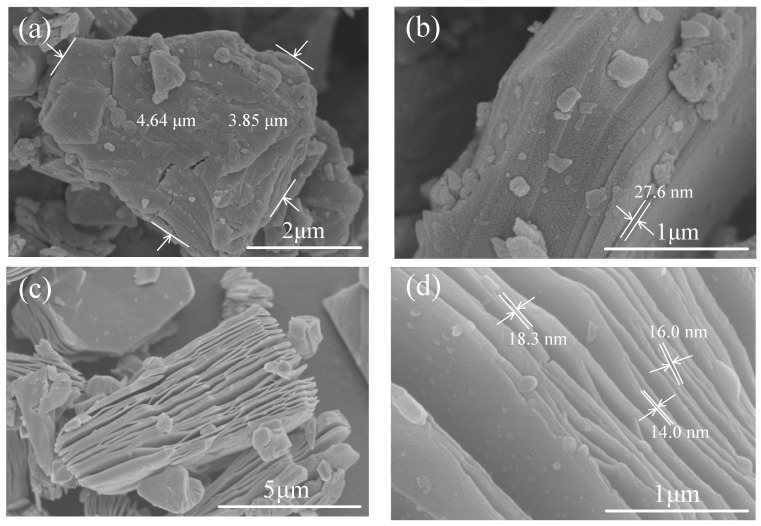
SEM images of Ti_3_AlC_2_ and Ti_3_C_2_T_x_. (**a**) Top view of Ti_3_AlC_2_ powder; (**b**) side view of Ti_3_AlC_2_ powder; (**c**) side views of Ti_3_C_2_T_x_ powder; and (**d**) enlarged side view of Ti_3_C_2_T_x_ powder with approximate thicknesses of layers.

Figure 2a shows a typical top view of Ti_3_AlC_2_ powders, with the lateral dimensions of around 4 to 5 μm. Figure 2b shows the side view of Ti_3_AlC_2_ powders, indicating the close-packed layer structure. After HF etching, the layers are separated and parallel to each other, as shown in Figure 2c. From Figure 2d, the thickness of the Ti_3_C_2_T_x_ layer was found to be around 10–20 nm. Such morphological transformations are similar to those observed in the literature [1,17], which indicates the selective removal of Al layers in Ti_3_AlC_2_.

**Figure 3:**
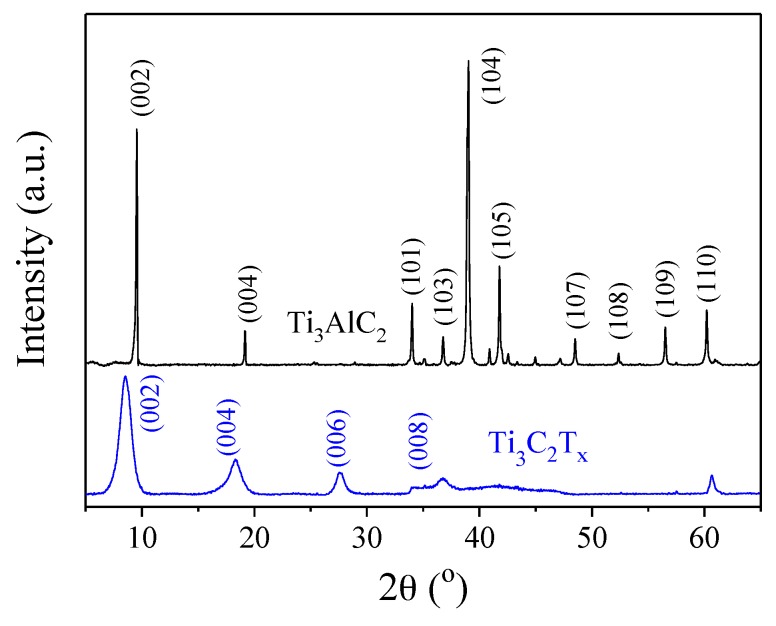
XRD results of Ti_3_AlC_2_ and Ti_3_C_2_T_x_.

To confirm the etching of Al, EDS and XRD were carried out on both Ti_3_AlC_2_ and Ti_3_C_2_T_x_ powders. From the EDS analysis, the content of Al significantly decreased and a very small amount of Al component was found in the Ti_3_C_2_T_x_. The removal of Al layer was also confirmed by the XRD results shown in Figure 3, where the characteristic (104) peak at 39° due to Al disappeared. Similar results were also reported in References [1,17]. Meanwhile, the (002) peak at 9.8° shifted to the lower angle, indicating the delamination of MXene layers.

#### 3.1.2. Ti_3_C_2_T_x_ Film

**Figure 4:**
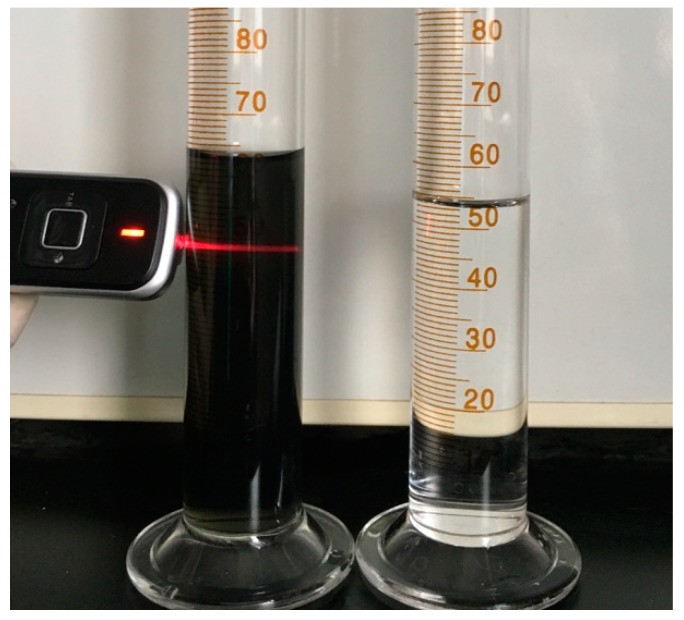
Colloidal dispersion of Ti_3_C_2_T_x_ nano-flakes (**left**) and distilled water (DI) water (**right**).

As described above, the Ti_3_C_2_T_x_ powders were delaminated into nano-flakes by the intercalation of DMSO and following ultrasonic treatment in water. The resulting black supernatant after centrifugation clearly demonstrated the Tyndall effect (Figure 4), i.e., scattering of laser light through the liquid, suggesting that the formation of the colloidal dispersion of delaminated Ti_3_C_2_T_x_.

**Figure 5:**
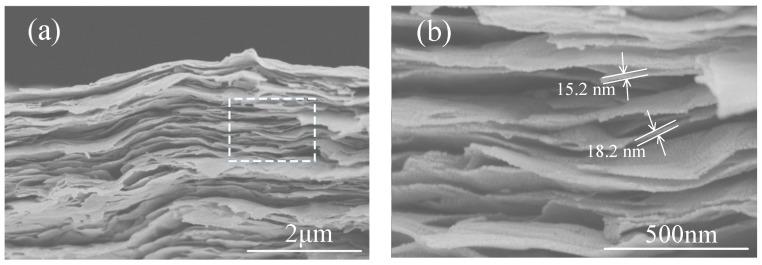
SEM images of (**a**) a cross-section of Ti_3_C_2_T_x_ film and (**b**) an enlarged part with estimated flake thickness.

Figure 5 shows the cross-sectional image of the Ti_3_C_2_T_x_ films, which were produced by vacuum filtration of the above colloidal dispersion. It was found that the as-prepared Ti_3_C_2_T_x_ film was composed of well-packed Ti_3_C_2_T_x_ nano-flakes with thicknesses of around 10–20 nm (Figure 5b), which are consistent with those results in Figure 2d. Small gaps exist between the flakes, which may affect both thermal and electrical conductivities of the Ti_3_C_2_T_x_ film, as will be discussed later.

### 3.2. Thermal and Electrical Conductivities of Ti_3_C_2_T_x_ Films

**Figure 6:**
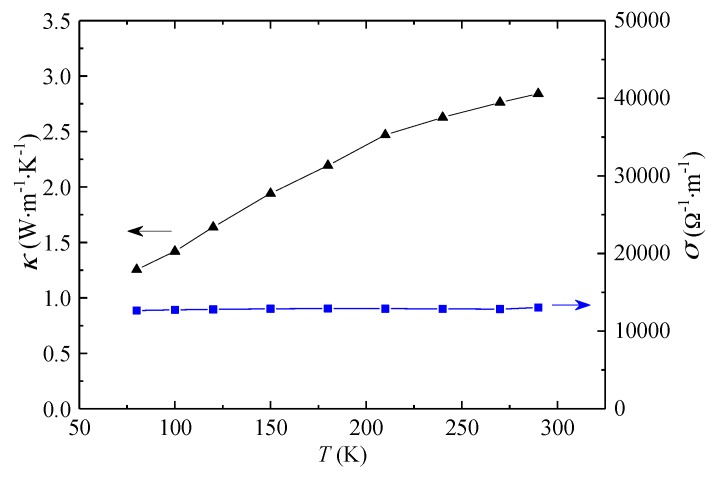
Thermal and electrical conductivities of Ti_3_C_2_T_x_ films.

As shown in Figure 6, the electrical conductivity of the Ti_3_C_2_T_x_ film remains nearly unchanged at ~12,800 Ω^−1^·m^−1^ when the temperature increases from 80 K to 290 K. These result are very close to that reported by Zhao et al. [18] (12,300 Ω^−1^·m^−1^) but lower than those from other works [6,19]. The difference in electrical conductivity could be related with the various amount of functional groups introduced to the surfaces of the 2D Ti_3_C_2_T_x_ flakes during the etching process [9]. In addition, the contact resistance at the junctions between the flakes could vary quite significantly depending on the preparation conditions of Ti_3_C_2_T_x_ films. In comparison, the thermal conductivity along the in-plane direction of the Ti_3_C_2_T_x_ film gradually increases from 1.26 W·m^−1^·K^−1^ at 80 K to 2.84 W·m^−1^·K^−1^ at 290 K.

Heat conduction in a solid sample could result from both electron and phonon transport. According to the Wiedemann-Franz law [20], the electron contribution to the thermal conductivity *κ_e_* is proportional to the electrical conductivity as

(2) $$\kappa_{e}=\sigma LT$$

where *σ* is the electrical conductivity, *L* is the Lorenz number which equals 2.45 × 10^−8^ W·Ω·K^−2^, and *T* is the absolute temperature. Based on the experimental results of the electrical conductivity in Figure 6, *κ_e_* can be calculated by Equation (2). The *κ_e_* is 0.025 W·m^−1^·K^−1^ at 80 K and 0.093 W·m^−1^·K^−1^ at 290 K, which accounts for only 1.98% and 3.26% of the measured thermal conductivity at the corresponding temperature.

Therefore, the increase of thermal conductivity with the temperature for the Ti_3_C_2_T_x_ films should be mainly attributed to the phonon transport. The relation between the thermal conductivity and phonon transport is widely acknowledged as [20]

(3) $$\kappa_{p}=\frac{1}{3}C\cdot \bar{v}\cdot l$$

where *C* is the heat capacity, $\bar{v}$ and *l* are the average group velocity, and mean free path of phonons, respectively. On the one hand, heat capacity increases with the increase of temperature. According to the Debye model [20], heat capacity becomes proportional to *T*^3^ when temperature *T* approaches 0 K, and it reaches a constant of 3R (R = 8.314 J·K^−1^·mol^−1^) when *T* is higher than the Debye temperature. Therefore, heat capacity generally increases when temperature increases from 80 K to 290 K. On the other hand, when temperature increases, the mean free path *l* decreases due to the increased phonon scattering. The combination of these two opposite effects eventually leads to an overall two-fold increase of thermal conductivity for the Ti_3_C_2_T_x_ films. Such phenomena was also observed in the thermal conductivity results of amorphous alloys [16].

### 3.3. Thermal Conductivity Analysis of Ti_3_C_2_T_x_ Films

The experimentally measured thermal conductivity is the effective in-plane thermal conductivity of the prepared Ti_3_C_2_T_x_ film that was composed of stacked delaminated Ti_3_C_2_T_x_ nano-flakes. Therefore, this effective thermal conductivity was determined by both the inherent thermal conductivity of the Ti_3_C_2_T_x_ flake and the inter-flake thermal resistance. Considering the periodical structure of the Ti_3_C_2_T_x_ film, one single Ti_3_C_2_T_x_ flake was selected as a unit cell with the same effective thermal conductivity as the Ti_3_C_2_T_x_ film. Applying the thermal resistance method [21,22], the relation between the effective and inherent thermal conductivities of the Ti_3_C_2_T_x_ flake can be expressed as

(4) $$\frac{D}{k_{eff}}=\frac{D}{k_{p}}+\sum R$$

where *k_eff_* and *k_p_* are the effective and inherent thermal conductivities of Ti_3_C_2_T_x_ flake, respectively, *D* is the characteristic lateral dimension of the Ti_3_C_2_T_x_ flake, and Σ*R* is the sum of inter-flake thermal resistance.

**Figure 7:**
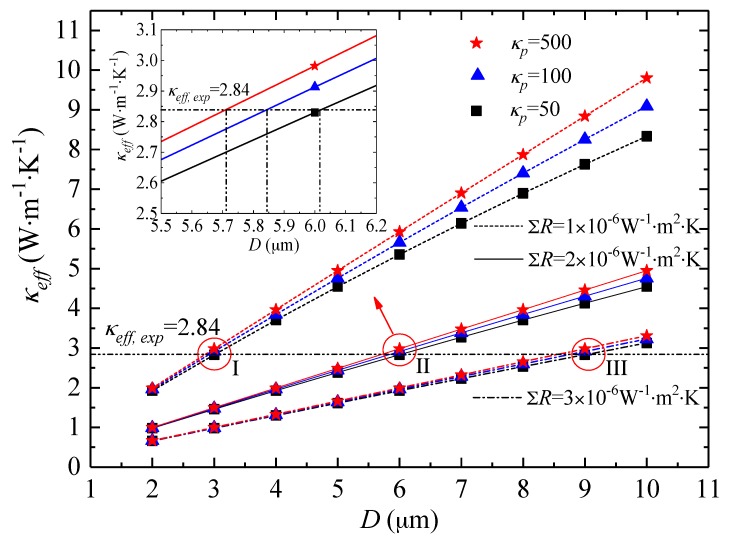
Evaluation of *k_eff_* of Ti_3_C_2_T_x_ film as a function of flake dimension, with certain preset flake thermal conductivity and inter-flake thermal resistance.

As we can see from Equation (4), if there is no gap and perfect contact exists between the Ti_3_C_2_T_x_ flakes, Σ*R* = 0 and thus *k_eff_ = k_p_*. However, in practice, Σ*R* is always larger than zero due to the presence of contact thermal resistance at the junctions between individual flakes and phonon scattering between adjacent flakes. According to Equation (4), the experimentally measured room temperature *k_eff_* of 2.84 W·m^−1^·K^−1^ for the Ti_3_C_2_T_x_ film may correspond to many possible combinations of *D*, *k_p_* and Σ*R*, as indicated by the circles of I, II, and III in Figure 7. However, the characteristic dimension *D*, which can be evaluated by SEM images, is no larger than the size of etched Ti_3_C_2_T_x_ flakes (<10 μm in this work). When *D* is determined, the combinations of the rest two parameters, i.e., the mathematically correlated parameters of *k_p_* and Σ*R*, are limited to a small range restricted by *k_eff_* and *D*. The three circles in Figure 7 show three combinations of *k_p_* and Σ*R*, with Σ*R* varying from 1 × 10^−6^ to 3 × 10^−6^ W^−1^·m^2^·K. To the best of our knowledge, the inherent thermal conductivity of Ti_3_C_2_T_x_ flake *k_p_* has not been experimentally determined in the literature. However, molecular simulation has been performed to predict the thermal conductivities of some MXene (Hf_2_CO_2_, Sc_2_CT_2_) nanosheets, to be from tens to hundreds W·m^−1^·K^−1^ [9]. Therefore, the inherent thermal conductity values of Ti_3_C_2_T_x_ flake, *k_p_*, used for the calculation in this work range from 50 to 500 W·m^−1^·K^−1^. Take the circle II for example, if the characteristic dimension *D* is around 6 μm, only the combination with Σ*R* of ~2 × 10^−6^ W^−1^·m^2^·K can yield a *k_eff_* of 2.84 W·m^−1^·K^−1^, as shown in the enlarged view of circle II in the insert of Figure 7. Therefore, with the help of model analysis, it is possible to correlate the main influencing parameters of the thermal conductivity measurement and to evaluate the overall thermal resistance that limits the thermal conductivity of the MXene film. It can also be found that among the three influencing parameters, the effects of Σ*R* and *D* on *k_eff_* of the Ti_3_C_2_T_x_ film are much more significant than *k_p_*.

Furthermore, the model results also provide guidance for manipulating the film thermal conductivity, which can be achieved by adjusting *D* and Σ*R*. The lateral dimension *D* of the MXene flake is mainly determined by the particle sizes of the original MAX materials and possible size reduction during the delamination processes including ultrasonic treatment. A larger *D* can lead to a higher film thermal conductivity when the other influencing parameters are kept constant. As for the overall thermal resistance Σ*R*, it mainly results from the contact resistance at the interfaces and phonon scattering between the adjacent Ti_3_C_2_T_x_ nano-flakes. Therefore, in order to improve the thermal conductivity of MXene film, measures should be taken to reduce Σ*R* by improving the interaction between the Ti_3_C_2_T_x_ nano-flakes. Recently, Liu et al. [10] manufactured Ti_3_C_2_T_x_ films by using multilayered Ti_3_C_2_T_x_ produced by selective etching of Al from Ti_3_AlC_2_. Comparing to the Ti_3_C_2_T_x_ film in this work, which is a stack of delaminated Ti_3_C_2_T_x_ nano-flakes, the Ti_3_C_2_T_x_ film in Liu’s work was made of un-delaminated multilayered Ti_3_C_2_T_x_ that retained more inherent connections between the layers (Figure 3 of Reference [10]). As a result, the reported thermal conductivity achieved 55.8 W·m^−1^·K^−1^ [10], which is an order of magnitude higher than the results in this work. This quite high film thermal conductivity also indicates that the inherent thermal conductivity of Ti_3_C_2_T_x_ flake should be higher than 55.8 W·m^−1^·K^−1^, which is consistent with the above analysis. In practice, due to the multiple requirements on physical properties, it is necessary to balance between multilayer and delamination structures, since delaminated nano-flakes can offer such advantages as high-surface areas and capacitance [6].

## 4. Conclusions

Ti_3_C_2_T_x_ nano-flakes were produced by selective Al etching of Ti_3_AlC_2_ and following DMSO intercalation and sonication-assisted delamination. Ti_3_C_2_T_x_ films composed of well-stacked Ti_3_C_2_T_x_ nano-flakes were prepared via a vacuum-assisted filtration. The state-of-the-art T-type method was applied to measure the thermal and electrical conductivities of the prepared film. Within the testing temperature range between 80 K and 290 K, the electrical conductivity of the Ti_3_C_2_T_x_ films remains almost constant at ~12,800 Ω^−1^·m^−1^. In comparison, the effective thermal conductivity of the same films increases from 1.26 W·m^−1^·K^−1^ at 80 K to 2.84 W·m^−1^·K^−1^ at 290 K. The experimental results of Ti_3_C_2_T_x_ MXene film thermal conductivity and Ti_3_C_2_T_x_ nano-flake dimension are correlated with the inherent thermal conductivity of Ti_3_C_2_T_x_ flakes and the thermal resistance between the stacked Ti_3_C_2_T_x_ flakes by a simplified thermal conductivity calculation model. The inherent thermal conductivity of the Ti_3_C_2_T_x_ flakes is estimated to be in the range of tens to hundreds W·m^−1^·K^−1^, and the thermal resistance between the stacked flakes is on the order of 10^−6^ W^−1^·m^2^·K.
